# Whole-Genome Characteristics and Polymorphic Analysis of Vietnamese Rice Landraces as a Comprehensive Information Resource for Marker-Assisted Selection

**DOI:** 10.1155/2017/9272363

**Published:** 2017-02-07

**Authors:** Hien Trinh, Khoa Truong Nguyen, Lam Van Nguyen, Huy Quang Pham, Can Thu Huong, Tran Dang Xuan, La Hoang Anh, Mario Caccamo, Sarah Ayling, Nguyen Thuy Diep, Cuong Nguyen, Khuat Huu Trung, Tran Dang Khanh

**Affiliations:** ^1^Laboratory of Bioinformatics, Institute of Biotechnology, Vietnam Academy of Science and Technology, 18 Hoang Quoc Viet, Cau Giay, Hanoi, Vietnam; ^2^Department of Genetic Engineering, Agricultural Genetics Institute, Vietnam Academy of Agricultural Sciences, Km2 Pham Van Dong, Tuliem, Hanoi, Vietnam; ^3^Graduate School for International Development and Cooperation, Hiroshima University, Hiroshima 739-8529, Japan; ^4^Genetics and Breeding, National Institute of Agricultural Botany, Cambridge, UK

## Abstract

Next generation sequencing technologies have provided numerous opportunities for application in the study of whole plant genomes. In this study, we present the sequencing and bioinformatic analyses of five typical rice landraces including three* indica* and two* japonica* with potential blast resistance. A total of 688.4 million 100 bp paired-end reads have yielded approximately 30-fold coverage to compare with the Nipponbare reference genome. Among them, a small number of reads were mapped to both chromosomes and organellar genomes. Over two million and eight hundred thousand single nucleotide polymorphisms (SNPs) and insertions and deletions (InDels) in* indica* and* japonica* lines have been determined, which potentially have significant impacts on multiple transcripts of genes. SNP deserts, contiguous SNP-low regions, were found on chromosomes 1, 4, and 5 of all genomes of rice examined. Based on the distribution of SNPs per 100 kilobase pairs, the phylogenetic relationships among the landraces have been constructed. This is the first step towards revealing several salient features of rice genomes in Vietnam and providing significant information resources to further marker-assisted selection (MAS) in rice breeding programs.

## 1. Introduction

Whole-genome sequencing (WGS) has revealed genetic information and genome structure and facilitated the identification of gene function of different plant species including some major crops such as rice, wheat, tomato, and soybean [1–5]. Next generation sequencing (NGS) methods are rapid and cost-effective, providing many promising applications for plant genomics and affording further insight into massive genomic variations [6, 7]. Combining NGS with bioinformatics is a powerful approach to detect DNA polymorphisms for quantitative trait loci (QTLs) analyses, marker-assisted selection, genome-wide association studies (GWAS), and linkage disequilibrium analysis in plants [8–11]. Moreover, DNA polymorphisms have been widely applied as DNA markers in genetic crop research [12].

Rice (*Oryza sativa* L.) is a staple crop and provides daily food for over half of the world's population. It contains two major groups,* indica* and* japonica*, which diverged more than one million years ago [13]. The recent sequencing of one representative from each group facilitated the study of the genomic structure of rice. In 2002, the first draft sequence of the* indica* genome was established by shotgun sequencing and homologous genes were predicted by comparison with the* Arabidopsis thaliana* genome by Yu et al. [14]. Subsequently, the complete genome sequence of* japonica* was generated in 2005 [15] and then updated using NGS and optical mapping in 2013 [1]. This genome version has been widely utilized as the reference rice genome for DNA polymorphism findings reported in some previous studies [16, 17].

Asian rice is one of the major worldwide cereal crops. Among Asian countries, Vietnam is one of the world's leading rice exporters, accounting for 16% of the world trade volume of rice [18]. To the best of our knowledge, the lack of whole-genome sequencing has raised concerns in relation to the characteristics of Vietnamese rice landraces; therefore, the objective of the current work was to perform genome analysis of five rice landraces, three* indica* and two* japonica*, collected in different ecological areas of Vietnam, by applying Illumina's paired-end sequencing. The generated reads were then mapped to the Nipponbare reference sequence to analyze the genomic features and discover and annotate candidate single nucleotide polymorphisms (SNPs) and insertions and deletions (InDels). The results presented may help to unravel the genetic basis of our rice genomes and polymorphic resources for molecular marker identification in the future.

## 2. Materials and Methods

### 2.1. Plant Materials and Whole-Genome Sequencing

The five Vietnamese rice landraces were collected from different ecological areas in Vietnam: Ha Giang (104°58′51′′E, 22^o^49′00′′N) and Lang Son (106°45′40′′E, 21°51′14′′N) in the northeast, Bac Ninh (106°04′24′′E, 21°11′15′′N) in the Red River Delta, Nghe An (104°58′38′′E, 19°10′35′′N) in the North Central Coast, and Can Tho (105°47′03′′E, 10°01′57′′N) in the Mekong River Delta. For convenience, the genomes were labeled (Table 1, Table S1 in the Supplementary Material available online at https://doi.org/10.1155/2017/9272363) as indicated in the list of Vietnamese native rice landraces according to the report of Trung and Ham [19]. Total DNA of each landrace was extracted from young leaf tissue using Qiagen DNeasy kit (Qiagen, Germany). The library preparation and sequencing of the rice genomes were carried out using Illumina HiSeq 2000 by applying Illumina pipeline 1.9 at the Genome Analysis Centre (TGAC), UK. The obtained FASTQ files were further assessed by FastQC software and deposited in the NCBI sequence read archive (SRA) with accession numbers SRP064171 (*indica* 12: SRR2529343,* indica* 13: SRR2543299,* indica* 15: SRR2543338,* japonica* 11: SRR2543336, and* japonica* 14: SRR2543337).

### 2.2. Mapping and Identification of SNPs and InDels

The paired-end reads were first aligned with the nuclear reference genome (*O. sativa* L. cv. Nipponbare, MSU release 7.0, GenBank accession PRJDB1747) and the organellar genomes (chloroplast genome, GenBank accession NC_001320.1; mitochondria genome, GenBank accession BA00029.3) using the alignment software BWA (version 0.6.2) with default parameters. The mapping quality was assessed by Qualimap (version 2.1) and BEDtools. The duplicated sequences were marked and removed by Picard tools (version 1.79). SNPs and InDels were then called and qualified by SAMtools (version 1.1) and VarScan (version 2.3.7) with the following parameters: mapping quality of 20, depth of coverage of 10, average of base quality of 30, and variant frequency of 0.1 with SNPs and 0.3 with InDels. The distribution of SNPs and InDels per 100 kb along the chromosome was used to determine SNP-poor regions (<1 SNP/1 kb) (Figure S1). Pearson correlation coefficients of SNP density among the landraces were calculated using R. The common reads mapped to both chromosomes and organelles were removed from BAM files using SAMtools (version 1.1) and BWA (version 0.6.2).

### 2.3. Annotation of SNPs and InDels

The SNPs and InDels were annotated using SnpEff (version 3.6) with the GFF file of the reference genome containing positional information of rice genomic regions, exons, 5′UTR, 3′UTR, and CDS. To identify outlier genes, the cutoff values of nonsynonymous SNPs were identified by using the five-number summary of box-and-whisker plot.

## 3. Results

### 3.1. Mapping of Whole-Genome Sequencing Reads

The whole genomes of three Vietnamese* indica* and two* japonica* rice landraces were resequenced and produced 688.4 million 100 bp paired-end reads in total. Among them, 621.8 million reads (90.32%) were successfully aligned to the Nipponbare nuclear reference genome. The alignment rates of each landrace were relatively high, ranging from 86.33% to 93.87%, yielding 30x–40x coverage in depth (Table 2) (Table S2a–e for chromosome coverage in detail).

The genome coverage ranged from 89.18% to 96.49% of the reference (Table 2 and Tables S2a–S2e). The organelle genomes (mitochondrial and chloroplast) had coverage of 94% −100% (Tables S3a and S3b). There was a portion of reads (0.17%~0.26%) that could be aligned to both nuclear DNA genome and organelle genomes, mitochondrial and chloroplast (Tables S4a–S4e).

### 3.2. Detection and Distribution of SNPs and InDels

**Figure 1:**
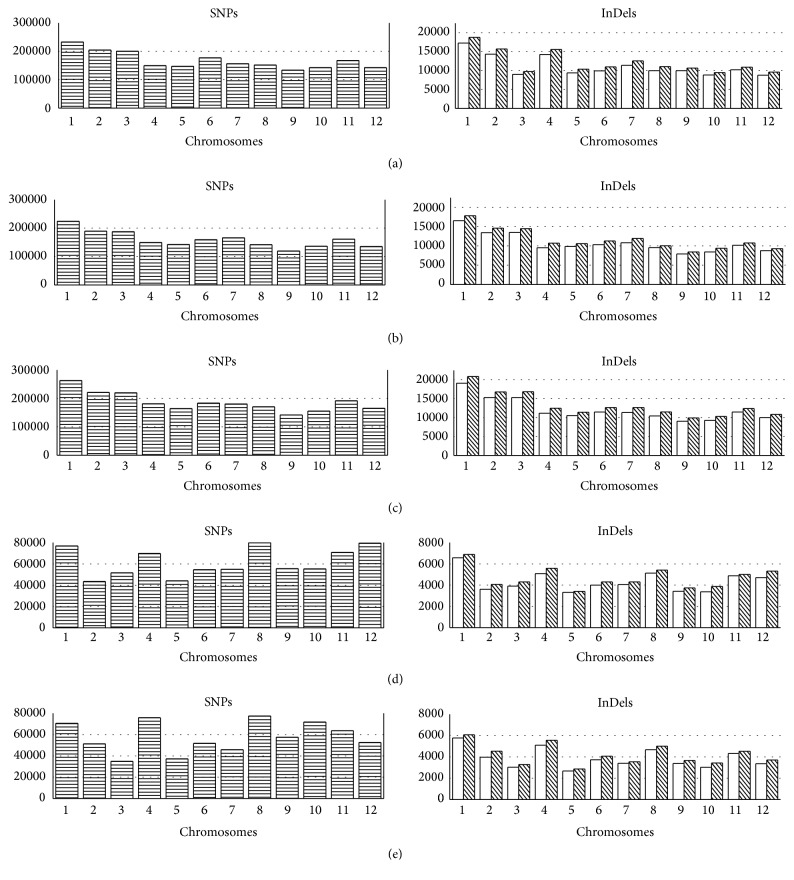
The number of SNPs and InDels on each chromosome in five landraces in comparison with the Nipponbare reference. The *x*-axis represents the chromosomes. The *y*-axis represents the number of SNPs (horizontal-line bars), insertions (white bars), and deletions (downward-diagonal bars). The five rice lines were (a)* indica* 12, (b)* indica* 13, (c)* indica* 15, (d)* japonica* 11, and (e)* japonica* 14.

**Figure 2:**
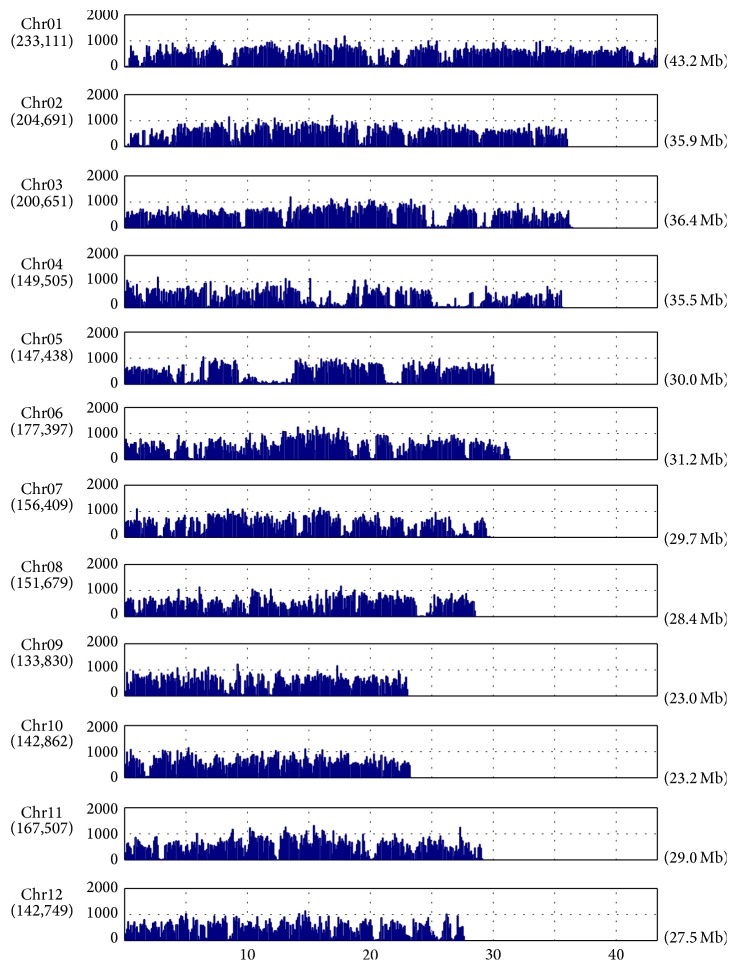
Distribution of SNPs between* indica *12 and Nipponbare on the 12 chromosomes. The *x*-axis shows the physical distance of chromosome into 100 kb windows. The chromosome size is indicated in brackets. The *y*-axis represents the number of SNPs per 100 kb. The total of SNPs in each chromosome is shown in parentheses.

By using SAMtools and VarScan software, the qualified SNPs and InDels were called. The distributions of SNPs and InDels by chromosomes of each landrace are shown in Figures 1 and 2.

For the* indica* lines, the numbers of SNPs and InDels were approximately two million and three hundred thousand, respectively (Tables S5a–S5c). Accordingly, for the* japonica* lines, the numbers of SNPs and InDels were approximately seven hundred thousand and one hundred thousand, respectively (Tables S5d and S5e). For the* indica* lines, chromosomes 1 and 9 had the highest and lowest variation rates in terms of both SNP and InDel, respectively. However, there was an exception in* indica* 12, and the lowest InDel rate was on chromosome 10 instead of chromosome 9 (Tables S5a–S5c). For the* japonica* lines, the highest SNP rate was on chromosome 8, while the lowest SNP rate was on chromosomes 2 (*japonica* 11) and 3 (*japonica* 14). The highest and lowest InDel rates were on chromosomes 1 and 5, respectively, for both (Tables S5d and S5e).

**Figure 3:**
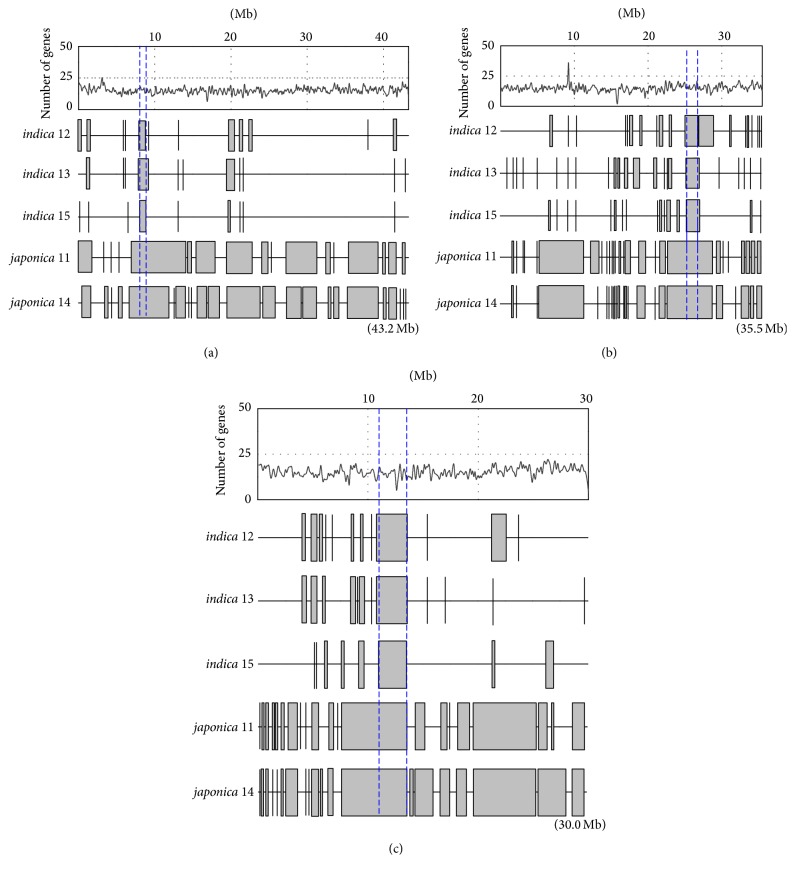
Distribution of “SNP deserts” on (a) chromosome 1, (b) chromosome 4, and (c) chromosome 5 in the five landraces. The low-SNP density regions are shown as gray bars or vertical lines. The vertical-dash intervals indicate the “SNP deserts” occurring in all five landraces. The line charts represent the distribution of genes across the chromosomes. The chromosome sizes are indicated in brackets.

**Figure 4:**
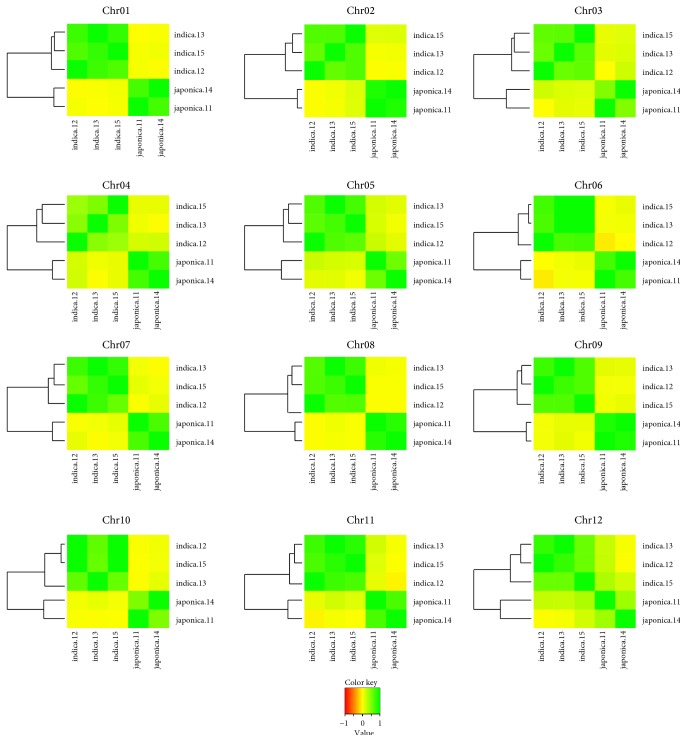
The heatmap and cluster view showing the correlation among the five landraces on the 12 chromosomes. Pearson correlation coefficients were calculated based on the SNP densities per 100 kb on the chromosome.

The distribution of DNA polymorphisms has been examined on each 100 kb nonoverlapping window to obtain average densities of SNP and InDels of chromosomes. The average densities of SNPs and InDels of* indica* landraces were about 2.5 times that of* japonica *landraces (Tables S5a–S5e). The average densities of deletions tended to be higher than those of insertions on all chromosomes (Tables S5a–S5e). Moreover, SNP deserts where the SNP densities were below 1 SNP/kb have been identified with differing sizes (100 kb to 6.7 Mb, average of 300 kb) and chromosomal locations. Of all landraces, there were three SNP deserts of larger sizes (2.6 MB, 0.8 MB, and 0.7 Mb) on chromosomes 5, 4, and 1, respectively (Figure 3). Moreover, within all five lines, there is a small SNP desert of 0.1 Mb located from position 12.4 to 12.5 Mb on chromosome 11 in which there are no genes found. We have used the SNP densities per 100 kb interval for reconstructing the relationships among the rice landraces. By calculating the Pearson correlation coefficient, the relationship between the rice lines was observed because the patterns grouped the rice landraces into two major subspecies, confirming the classification of landraces based on the chloroplast DNA presence/absence of a deletion in the Pst-12 fragment [19]. In detail, the landraces in the same subspecies had a positive correlation close to one, whereas there was no linear relationship between* indica* and* japonica* lines with the coefficients around zero (Figure 4).

### 3.3. Annotation of SNPs and InDels

**Figure 5:**
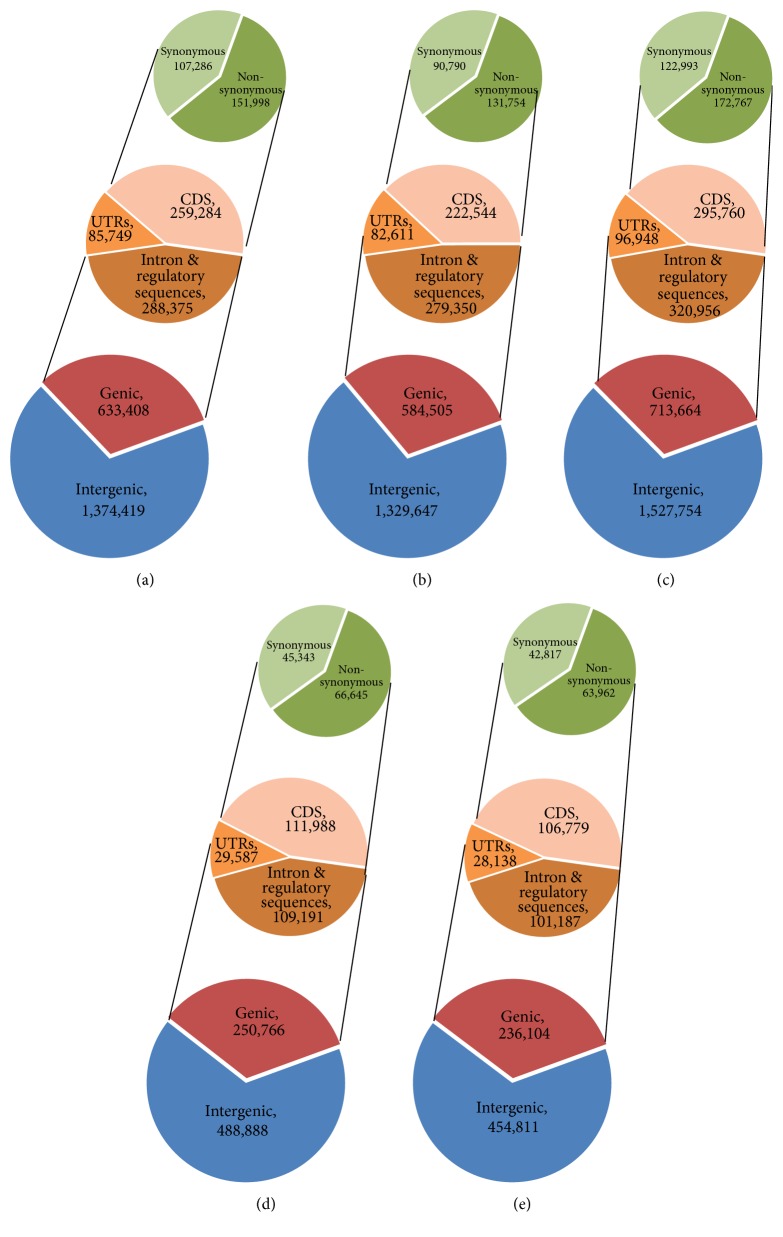
Annotation of single nucleotide polymorphisms (SNPs) between five Vietnamese rice landraces ((a)* indica *12, (b)* indica *13, (c)* indica* 15, (d)* japonica* 11, and (e)* japonica* 14) and Nipponbare based on the annotations of Nipponbare reference genome.

SNPs and InDels were annotated against the GFF file of the Nipponbare reference genome using SnpEff. SNPs mostly occurred in the intergenic regions (approximately 68.0% for* indica*, 66.0% for* japonica*), respectively (Figure 5, Table 3). For SNPs within genic regions (approximately 32.0% for* indica*, 34.0% for* japonica*), SNPs occurred in introns and regulatory sequences (44.9% to 47.8% for* indica*, about 43.0% for* japonica*) and UTR regions (approximately 13.75% for* indica* and 12% for* japonica*).

**Figure 6:**
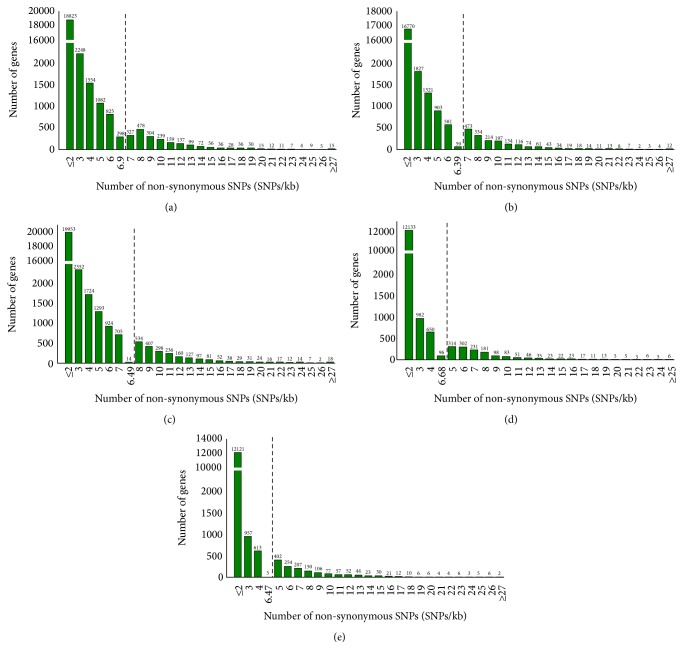
The distribution and skewness of the nonsynonymous SNPs. SNPs per 1 kb in (a)* indica* 12, (b)* indica* 13, (c)* indica* 15, (d)* japonica* 11, and (e)* japonica* 14. The black dash indicates the outlier value cutoff.

Among CDS regions, the split between nonsynonymous and synonymous SNPs was 58.75%, 41.25% for* indica*, and 59.7%, 40.3% for* japonica* (Figure 5, Table 3). Similarly, of the InDels, more than 73.0% were detected in intergenic regions. Most of the InDels within genic regions were within InDels or regulatory sequences (more than 62.0%), with 9.33% to 11.95% within coding sequences (Figure S2). The length of insertions ranged from one to 27 bp while the length of deletions detected was up to 41 bp. The majority of InDels were mononucleotide (≈55.5%) and dinucleotide (≈16.65%). In order to provide more insights into the effects of nonsynonymous SNPs on the genes, the distribution and skewness were calculated to identify outlier genes, which possess very high numbers of nonsynonymous SNPs as shown in Figure 6. According to the Nipponbare reference genome, nearly half of the 149 outlier genes were retrotransposon proteins (Table S6).

We have also observed that the number of transitional SNPs (A/G and C/T) was much higher than that of transversions (A/C, A/T, C/G, and G/T) for all of the five landraces with the ratios (Ts/Tv) ranging from 2.19 (*japonica* 14) to 2.37 (*indica* 12). Within transitions, the frequency of C/T was slightly higher than those of A/T and much larger than transversion SNPs (Table 4).

## 4. Discussion

In this study, we have sequenced five Vietnamese rice genomes consisting of three* indica* and two* japonica* landraces. The sequence datasets were aligned to the Nipponbare reference genome to identify genetic variations. The genetic variation annotation and analysis provided novel insights into the specific Vietnamese rice landraces which should be a good resource for further molecular breeding in rice. We have analyzed the sequence datasets of two major groups of rice landraces,* indica* and* japonica*, through five elite landraces in Vietnam. This provides significant information as to the genetic diversity of the two types of rice lines in the domestication process. The results have further contributed additional evidence for the transfer of DNA regions in the organelle into the nucleus in the rice genome. Our study has also strengthened the classification of the relationship among the landraces. The detection of DNA polymorphisms can be used to identify novel genes that differentiated between our landraces and will serve as a reference for studies relating to specialty characteristics of Vietnamese rice landraces. These polymorphisms are also available for use in marker-assisted selection in rice breeding programs.

The whole genomes of five rice landraces have yielded high-quality reads, from 112 to 161 million reads per line. Most of the reads (≥86.33%) were successfully mapped to the Nipponbare reference genome with the breadth of coverage of more than 89.18%, proving that the selected rice genomes are similar to the reference. Interestingly, we found a small number of reads aligned with both chromosomes and organelle genomes including chloroplast and mitochondria. This phenomenon is known as “organellar insertion” in the nuclear genome, which has been previously reported by the International Rice Genome Sequencing Project [20]. The reads mapped to the mitochondria concentrated on chromosome 12 (1.0%), and the reads mapped to the chloroplast located on chromosome 10 (0.8%). Therefore, using NGS, these data are consistent with some previous reports and also reconfirmed that chromosomes 10 and 12 have had more insertions than the others [20].

The sequencing depth of approximately 30x has been sufficient for detecting DNA polymorphisms. Our results of variant calling of* indica* lines (1,914,152 to 2,241,418 SNPs, 268,400 to 303,039 InDels) are in agreement with the previous study reporting that the 93-11* indica* possesses about 1.7 million SNPs and 480 thousand InDels [21]. However, the numbers of SNPs and InDels (about 714 thousand SNPs and 102 thousand InDels) of* japonica* lines were about five and three times those of Omachi line (132,462 SNPs and 35,766 InDels) but were similar to those of Moroberekan (827,448 SNPs and 159,597 InDels), a tropical* japonica* cultivar [12, 22]. The distinct difference between tropical and temperate* japonica* landraces has been reported by Arai-Kichise et al. [22]. Further validation is underway to obtain SNP markers for rice breeding selection.

SNP deserts, genome regions of SNP rate less than 1 SNP/kb, were identified in all 5 Vietnamese landraces with sizes varying from 100 kb to 6.7 MB. The* japonica* SNP deserts are longer than those of* indica*. Mostly, SNP deserts have been previously found on chromosome 5 [23, 24]; however, we have identified two additional SNP deserts within all five Vietnamese landraces with the size of 0.7 Mb on chromosome 1 and 0.8 Mb on chromosome 4. Cheng et al. [25] have reported that SNP deserts were in the vicinity of the centromere on chromosome 5 but far from the centromere on chromosomes 1 and 4. Therefore, the current results have supported the hypothesis that SNP-low regions have not been correlated with low recombination [26]. The common SNP deserts might include highly conserved regions among the five Vietnamese rice landraces. They could result from selective sweeps reducing the variants during human selection and rice domestication [27]. These SNP deserts are able to raise fascinating questions for future studies as to whether the persistence of chromosomal regions is random or special for the individual landrace.

The SNP distribution correlation analysis of the five landraces has demonstrated the distinct divergence between* indica* and* japonica *and disclosed the phylogenetic relationships among the landraces (Figure 4); thus, it is possible to be exploited for the verification of landrace classifications. The relationship among the rice landraces in the correlation of chromosomes could be utilized for the genetic linkage disequilibrium studies.

Additionally, the phenomenon “transition bias,” which means the ratios of transitions (Ts) and transversions (Tv) larger than 1 : 2, occurred among the five landraces. The transitional SNPs are more tolerated than transversional ones during mutation and natural selection because they are more likely synonymous in coding protein, resulting in conserving the protein structure [28]. Within transitions, a number of both A/G and C/T changes have been rather similar. Among transversions, the A/T transversions have been shown to be higher than others which was also reported in the genomes of citrus and rice [17, 28, 29].

In summary, by sequencing and aligning five Vietnamese rice genomes with the reference genome, Nipponbare, our results have disclosed interesting genetic information such as SNP and InDel distributions and effects and SNP deserts. Three “SNP desert” regions, which might result from selective sweeps in the domestication of rice landraces, were also observed in the different chromosomes. Furthermore, the SNP distribution analysis has revealed the phylogeny of* indica *and* japonica *with distinct classification. Further SNP validations need to be examined to identify accurate SNP markers for molecular breeding.

## Supplementary Material

The supplementary material contains:
(1) Distribution of SNPs between indica 13, indica 15, japonica 11 and Nipponbare on the 12 chromosomes (Figure S1a; Figure S1b; Figure S1c);
(2) Annotation of InDels between five Vietnamese rice cultivars (A: indica 12; B: indica 13; C: indica 15; D: japonica 11; E: japonica 14) and Nipponbare (Figure S2);
(3) The morphological characteristics of five Vietnamese landraces (Table S1);
(4) Coverage of the reads from indica 12, indica 13, indica 15, japonica 11, japonica 14 to nuclear Nipponbare reference genome (Table S2a, Table S2b, Table S2c, Table S2d, Table S2e);
(5) Mapping and coverage of the reads from landraces to mitochondrial Nipponbare reference genome; (Genbank accession: BA00029.3; Genbank accession: NC_001320.1) (Table S3a, Table S3b);
(6) The number of common reads mapped to both chromosome and organelle of the reference genome in indica 12, indica 13, indica 15, japonica 11, japonica 14 (Table S4a, Table S4b, Table S4c, Table S4d, Table S4e);
(7) Polymorphisms of indica 12, indica 13, indica 15, japonica 11, japonica 14 genome compared to Nipponbare reference (Table S5a, Table S5b, Table S5c, Table S5d, Table S5e);
(8) List of high nsSNPs shared in the five Vietnamese landraces (Table S6).

## Figures and Tables

**Table 1:** Abbreviation list of Vietnamese rice genomes used in the study.

Abbreviation	Name of rice landraces (name in Vietnamese)	Subspecies	Origin
*indica* 12	Chiem nho Bac Ninh 2	*indica *	Bac Ninh province
*indica* 13	Nep lun	*indica*	Ha Giang province
*indica* 15	OM6377	*indica*	Can Tho province
*japonica* 11	Bletelo	*japonica*	Lang Son province
*japonica* 14	Khau mac buoc	*japonica*	Nghe An province

**Table 2:** Mapping and coverage of the reads from the landraces to Nipponbare reference genome.

Landraces	*indica *12	*indica *13	*indica* 15	*japonica* 11	*japonica *14
Total reads^a^	129,251,696	112,867,645	151,576,754	133,602,832	161,141,384
# mapped reads^b^	112,667,155	101,814,936	130,851,417	125,253,671	151,261,159
Mapped reads (%)	87.17%	90.21%	86.33%	93.75%	93.87%
# unmapped reads^c^	16,584,541	11,052,709	20,725,337	8,349,161	9,880,225
Unmapped reads (%)	12.83%	9.79%	13.67%	6.25%	6.13%
Mean mapping quality^d^	42.21	44.59	42.03	47.81	48.14
Coverage^e^	91.30%	89.18%	92.56%	95.50%	96.49%
Depth^f^	29.94	27.09	34.74	33.46	40.41

^a^The raw reads in the FASTQ files. ^b^The number of reads mapped to 12 chromosomes in the nucleus. ^c^The number of unmapped reads to both nuclear and organellar genomes. ^d^The error probability of read mapping scaled by Phred quality. ^e^The breadth of coverage across the nuclear genome. ^f^The sequencing depth of reads.

**Table 3:** Number of SNP effects annotated by SnpEff. SNPs were classified into various genomic regions (intergenic regions, intron, and exon) and effect terms by types. The total of intergenic, intronic, and exonic SNPs was more than the total number of SNPs due to overlapping gene models in the GFF file.

	*indica* 12		*indica* 13		*indica *15		*japonica* 11		*japonica* 14	
Intergenic	1,374,419	68.45	1,329,647	69.46	1,527,754	68.16	488,888	66.10	454,811	65.83
Genic	633,408	31.55	584,505	30.54	713,664	31.84	250,766	33.90	236,104	34.17
Intron & regulatory sequences	288,375	45.53	279,350	47.79	320,956	44.97	109,191	43.54	101,187	42.86
UTRs	85,749	13.54	82,611	14.13	96,948	13.58	29,587	11.80	28,138	11.92
CDS	259,284	40.93	222,544	38.07	295,760	41.44	111,988	44.66	106,779	45.23
Nonsynonymous	151,998	58.62	131,754	59.20	172,767	58.41	66,645	59.51	63,962	59.90
Synonymous	107,286	41.38	90,790	40.80	122,993	41.59	45,343	40.49	42,817	40.10

**Table 4:** Base changes in five rice genomes.

Landraces	*indica *12	*indica* 13	*indica *15	*japonica* 11	*japonica* 14
Transitions					
A/G	705,908	671,815	787,002	255,473	236,847
C/T	707,496	672,959	787,976	255,542	237,620
Transversions					
A/C	155,310	149,265	174,014	59,111	55,410
A/T	182,882	176,243	205,343	70,949	68,165
G/C	101,541	95,523	113,736	39,460	37,120
G/T	154,639	148,304	173,286	59,011	55,639
Ts/Tv	2.378	2.362	2.364	2.236	2.193
