# Supplementary material for: Whole-Genome Characteristics and Polymorphic Analysis of Vietnamese Rice Landraces as a Comprehensive Information Resource for Marker-Assisted Selection
Source: Int J Genomics. 2017 Feb 7;2017:9272363. doi: 10.1155/2017/9272363 (PMC5318636; doi:10.1155/2017/9272363)
Supplement: Supplementary file 1 — The supplementary material contains: (1) Distribution of SNPs between indica 13, indica 15, japonica 11 and Nipponbare on the 12 chromosomes (Figure S1a; Figure S1b; Figure S1c); (2) Annotation of InDels between five Vietnamese rice cultivars (A: indica 12; B: indica 13; C: indica 15; D: japonica 11; E: japonica 14) and Nipponbare (Figure S2); (3) The morphological characteristics of five Vietnamese landraces (Table S1); (4) Coverage of the reads from indica 12, indica 13, indica 15, japonica 11, japonica 14 to nuclear Nipponbare reference genome (Table S2a, Table S2b, Table S2c, Table S2d, Table S2e); (5) Mapping and coverage of the reads from landraces to mitochondrial Nipponbare reference genome; (Genbank accession: BA00029.3; Genbank accession: NC_001320.1) (Table S3a, Table S3b); (6) The number of common reads mapped to both chromosome and organelle of the reference genome in indica 12, indica 13, indica 15, japonica 11, japonica 14 (Table S4a, Table S4b, Table S4c, Table S4d, Table S4e); (7) Polymorphisms of indica 12, indica 13, indica 15, japonica 11, japonica 14 genome compared to Nipponbare reference (Table S5a, Table S5b, Table S5c, Table S5d, Table S5e); (8) List of high nsSNPs shared in the five Vietnamese landraces (Table S6). [file 9272363.f1.docx]

**Supplementary Materials**

The supplementary material contains: (1) Distribution of SNPs between *indica* 13, *indica* 15, *japonica* 11 and Nipponbare on the 12 chromosomes (Figure S1a; Figure S1b; Figure S1c); (2) Annotation of InDels between five Vietnamese rice cultivars (A: *indica* 12*;* B: *indica* 13; C: *indica* 15; D: *japonica* 11; E: *japonica* 14) and Nipponbare (Figure S2); (3) The morphological characteristics of five Vietnamese landraces (Table S1); (4) Coverage of the reads from *indica* 12, *indica* 13, *indica* 15, *japonica* 11, *japonica* 14 to nuclear Nipponbare reference genome (Table S2a, Table S2b, Table S2c, Table S2d, Table S2e); (5) Mapping and coverage of the reads from landraces to mitochondrial Nipponbare reference genome; (Genbank accession: BA00029.3; Genbank accession: NC_001320.1) (Table S3a, Table S3b); (6) The number of common reads mapped to both chromosome and organelle of the reference genome in *indica* 12, *indica* 13, *indica* 15, *japonica* 11, *japonica* 14 (Table S4a, Table S4b, Table S4c, Table S4d, Table S4e); (7) Polymorphisms of *indica* 12, *indica* 13, *indica* 15, *japonica* 11, *japonica* 14 genome compared to Nipponbare reference (Table S5a, Table S5b, Table S5c, Table S5d, Table S5e); (8) List of high nsSNPs shared in the five Vietnamese landraces (Table S6)

Figure S1a. Distribution of SNPs between *indica* 13 and Nipponbare on the 12 chromosomes. The x-axis shows the physical distance along the chromosome in 100-kb windows. The chromosome size is indicated in brackets. The y-axis represents the number of SNPs per 100kb. The total number of SNPs in each chromosome is shown in parentheses.

Figure S1b. Distribution of SNPs between *indica* 15 and Nipponbare on the 12 chromosomes. The x-axis showed the physical distance of chromosome into 100-kb windows. The chromosome size was indicated in brackets. The y-axis represented the number of SNPs per 100kb. The total of SNPs in each chromosome was shown in parentheses.

Figure S1c. Distribution of SNPs between *japonica* 11 and Nipponbare on the 12 chromosomes. The x-axis shows the physical distance along the chromosome in 100-kb windows. The chromosome size is indicated in brackets. The y-axis represents the number of SNPs per 100kb. The total number of SNPs in each chromosome is shown in parentheses.

Figure S1d. Distribution of SNPs between *japonica* 14 and Nipponbare in the 12 chromosomes. The x-axis shows the physical distance along the chromosome in 100-kb windows. The chromosome size is indicated in brackets. The y-axis represents the number of SNPs per 100kb. The total number of SNPs in each chromosome is shown in parentheses.


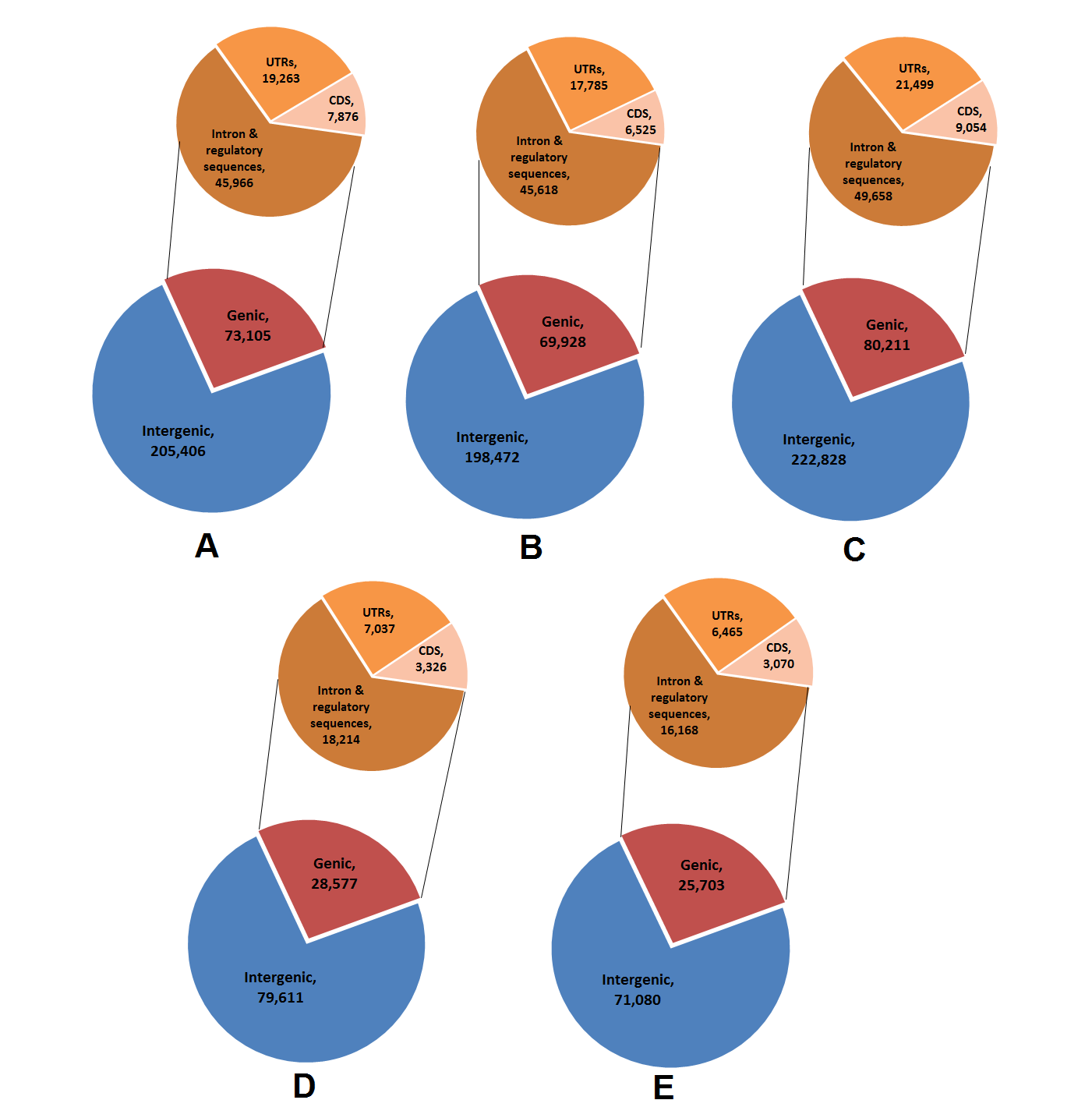


Figure S2. Annotation of InDels between five Vietnamese rice cultivars (A: *indica* 12*;* B: *indica* 13; C: *indica* 15; D: *japonica* 11; E: *japonica* 14) and Nipponbare based on the annotations of Nipponbare reference genome.

Table S1. The morphological characteristics of five Vietnamese landraces

|  | Morphological characteristics | *indica* 12 | *indica* 13 | *indica* 15 | *japonica* 11 | *japonica* 14 |
| --- | --- | --- | --- | --- | --- | --- |
| 1 | Seeding Height (SH) | 26,4 (cm) | 24,7 (cm) | 35,2 (cm) | 49,2 (cm) | 45,5 (cm) |
| 2 | Leaf Length (LL) | 50,6 (cm) | 34,8 (cm) | 38,9 (cm) | 49,2 (cm) | 51,4 (cm) |
| 2 | Leaf Width (LW) | 0,88 (cm) | 1,26 (cm) | 1,48 (cm) | 1,12 (cm) | 1,20 (cm) |
| 3 | Leaf Blade Pubescence (LBP) | Pubescent | Intermediate | Intermediate | Intermediate | Glabrous |
| 4 | Leaf Blade Color (LBC) | Green | Green | Green | Green | Dark Green |
| 5 | Basal Leaf Sheath Color (BLSC) | Green | Green | Green | Green | Green |
| 6 | Leaf Angle (LA) | Horizontal | Erect | Erect | Horizontal | Horizontal |
| 7 | Flag Leaf Angle (FLA) | Intermediate | Erect | Erect | Descending | Descending |
| 8 | Ligule Lenght (LgL) | 1,24 (cm) | 2,40 (cm) | 1,65 (cm) | 1,96 (cm) | 1,44 (cm) |
| 9 | Ligule Color (LgC) | White | White | White | White | White |
| 10 | Ligule Shape (LS) | Cleft | Cleft | Acute to acuminate | Cleft | Acute to acuminate |
| 11 | Collar Color (CC) | Light Green | Light Green | Light Green | Light Green | Light Green |
| 12 | Auricle Color (AC) | Light Green | Light Green | Light Green | Light Green | Light Green |
| 13 | Culm Length (Cml) | 117,2 (cm) | 60,2 (cm) | 85,0 (cm) | 63,0 (cm) | 89,8 (cm) |
| 14 | Culm Number (CmN) | 11,2 | 4,0 | 12,0 | 5,4 | 5,0 |
| 15 | Culm Angle (CmA) | Open, = 60^0^ | Erect, < 30^0^ | Erect, < 30^0^ | Intermediate, = 45^0^ | Intermediate, = 45^0^ |
| 16 | Diameter of Basal Internode (DBI) | 3,00 (mm) | 4,60 (mm) | 7,00 (mm) | 4,17 (mm) | 4,30 (mm) |
| 17 | Culm Internode Color (CmIC) | Light gold | Green | Light gold | Light gold | Light gold |
| 18 | Panicle Length (PnL) | 26,0 (cm) | 23,4 (cm) | 22,5 (cm) | 24,6 (cm) | 25,0 (cm) |
| 19 | Panicle Type (PnT) | Open | Compact | Compact | Intermediate | Open |
| 20 | Secondary Branching of Panicle (PnBr) | Heavy | Heavy | Heavy | Clustered | Light |
| 21 | Panicle Axis (PnA) | Droopy | Droopy | Droopy | Droopy | Droopy |
| 22 | Awning (An) | Absent | Short and partly awned | Short and partly awned | Absent | Absent |
| 23 | Awn Color (AnC) | Absent | Straw | Straw | Absent | Absent |
| 24 | Apiculus Color (ApC) | Straw | Straw | Straw | Brown | Brown |
| 25 | Stigma Color (SgC) | White | White | White | White | White |
| 26 | Lemma and Palea Color (LmPC) | Gold and gold furrows on straw background | Straw | Straw | Gold and gold furrows on straw background | Brown spots on straw |
| 27 | Lemma and Palea Pubescence (LmPb) | Long hairs (velvety) | Short hairs | Glabrous | Glabrous | Glabrous |
| 28 | Sterile Lemma Color (SLmC) | Gold | Gold | Straw (yellow) | Gold | Red |
| 29 | Sterile Lemma Length (SLmL) | Medium, (1,6-2,5 mm) | Medium, (1,6-2,5 mm) | Medium, (1,6-2,5 mm) | Long, > 2,5 mm | Long, > 2,5 mm |
| 30 | Grain Lenght (GrL) | 8,37 (mm) | 9,34 (mm) | 10,92 (mm) | 10,28 (mm) | 8,71 (mm) |
| 31 | Grain Width (GrW) | 2,57 (mm) | 3,29 (mm) | 2,21 (mm) | 3,56 (mm) | 3,75 (mm) |

Table S2a. Coverage of the reads from *indica* 12 to nuclear Nipponbare reference genome

|  | Chromosome length (bp)^a^ | Aligned length (bp)^b^ | Coverage in breadth (%)^c^ | Depth^d^ |
| --- | --- | --- | --- | --- |
| Chromosome 1 | 43,270,923 | 40,181,379 | 92.86 | 28.94 |
| Chromosome 2 | 35,937,250 | 33,392,893 | 92.92 | 29.09 |
| Chromosome 3 | 36,413,819 | 34,786,121 | 95.53 | 29.83 |
| Chromosome 4 | 35,502,694 | 32,140,589 | 90.53 | 31.57 |
| Chromosome 5 | 29,958,434 | 28,502,454 | 95.14 | 32.12 |
| Chromosome 6 | 31,248,787 | 28,502,019 | 91.21 | 28.96 |
| Chromosome 7 | 29,697,621 | 27,015,926 | 90.97 | 28.84 |
| Chromosome 8 | 28,443,022 | 25,661,294 | 90.22 | 29.59 |
| Chromosome 9 | 23,012,720 | 20,817,307 | 90.46 | 30.73 |
| Chromosome 10 | 23,207,287 | 20,861,030 | 89.89 | 35.39 |
| Chromosome 11 | 29,021,106 | 25,062,627 | 86.36 | 26.36 |
| Chromosome 12 | 27,531,856 | 23,842,587 | 86.6 | 29.44 |
| Total/Average | 373,245,519 | 340,766,226 | 91.3 | 29.94 |
| ^a^Length of Nipponbare refenrence genome (Genbank accession - PRJDB1747). | | |  |  |
| ^b^Length of consensus sequence. | |  |  |  |
| ^c^Coverage with locations containing at least one read. | |  |  |  |
| ^d^Sequencing depth of reads. | |  |  |  |

| Table S2b. Coverage of the reads from *indica* 13 to nuclear Nipponbare reference genome | | | |  |
| --- | --- | --- | --- | --- |
| Chromosome | Chromosome length (bp) ^a^ | Aligned length (bp) ^b^ | Coverage in breadth (%)^c^ | Depth^d^ |
| Chromosome 1 | 43,270,923 | 39,380,867 | 91.01 | 26.24 |
| Chromosome 2 | 35,937,250 | 32,706,491 | 92.16 | 26.9 |
| Chromosome 3 | 36,413,819 | 33,140,217 | 93.85 | 26.82 |
| Chromosome 4 | 35,502,694 | 32,311,002 | 87.93 | 28.06 |
| Chromosome 5 | 29,958,434 | 27,265,171 | 93.38 | 28.77 |
| Chromosome 6 | 31,248,787 | 28,439,521 | 89.87 | 26.53 |
| Chromosome 7 | 29,697,621 | 27,027,805 | 87.86 | 25.54 |
| Chromosome 8 | 28,443,022 | 25,885,994 | 87.64 | 26.72 |
| Chromosome 9 | 23,012,720 | 20,943,876 | 88.42 | 28.27 |
| Chromosome 10 | 23,207,287 | 21,120,952 | 88.14 | 32.2 |
| Chromosome 11 | 29,021,106 | 26,412,109 | 84.02 | 23.43 |
| Chromosome 12 | 27,531,856 | 25,056,742 | 82.38 | 27.14 |
| Total/Average | 373,245,519 | 339,690,747 | 89.18 | 27.09 |
| ^a^Length of Nipponbare refenrence genome (Genbank accession - PRJDB1747). | | |  |  |
| ^b^Length of consensus sequence. | |  |  |  |
| ^c^Coverage with locations containing at least one read. | |  |  |  |
| ^d^Sequencing depth of reads. | |  |  |  |

| Table S2c: Coverage of the reads from *indica* 15 to nuclear Nipponbare reference genome | | | | |
| --- | --- | --- | --- | --- |
| Chromosome | Chromosome length (bp) ^a^ | Aligned length (bp) ^b^ | Coverage in breadth (%)^c^ | Depth^d^ |
| Chromosome 1 | 43,270,923 | 40,813,135 | 94.32 | 33.87 |
| Chromosome 2 | 35,937,250 | 33,896,014 | 94.51 | 34.64 |
| Chromosome 3 | 36,413,819 | 34,345,514 | 95.98 | 34.99 |
| Chromosome 4 | 35,502,694 | 33,486,141 | 91.52 | 34.96 |
| Chromosome 5 | 29,958,434 | 28,256,795 | 95.81 | 37.47 |
| Chromosome 6 | 31,248,787 | 29,473,856 | 92.83 | 34.43 |
| Chromosome 7 | 29,697,621 | 28,010,796 | 90.49 | 32.72 |
| Chromosome 8 | 28,443,022 | 26,827,458 | 92.73 | 35.39 |
| Chromosome 9 | 23,012,720 | 21,705,598 | 92.25 | 34.82 |
| Chromosome 10 | 23,207,287 | 21,889,113 | 91.38 | 40.69 |
| Chromosome 11 | 29,021,106 | 27,372,707 | 87.79 | 29.71 |
| Chromosome 12 | 27,531,856 | 25,968,047 | 88.6 | 34.79 |
| Total/Average | 373,245,519 | 352,045,174 | 92.56 | 34.74 |
| ^a^Length of Nipponbare refenrence genome (Genbank accession - PRJDB1747). | | |  |  |
| ^b^Length of consensus sequence. | |  |  |  |
| ^c^Coverage with locations containing at least one read. | |  |  |  |
| ^d^Sequencing depth of reads. | |  |  |  |

| Table S2d: Coverage of the reads from *japonica* 11 to nuclear Nipponbare reference genome | | | | |
| --- | --- | --- | --- | --- |
| Chromosome | Chromosome length (bp) ^a^ | Aligned length (bp) ^b^ | Coverage in breadth (%)^c^ | Depth^d^ |
| Chromosome 1 | 43,270,923 | 41,938,179 | 96.92 | 33.56 |
| Chromosome 2 | 35,937,250 | 35,128,662 | 97.75 | 34.21 |
| Chromosome 3 | 36,413,819 | 35,780,219 | 98.26 | 33.33 |
| Chromosome 4 | 35,502,694 | 33,599,750 | 94.64 | 34.53 |
| Chromosome 5 | 29,958,434 | 29,203,481 | 97.48 | 33.63 |
| Chromosome 6 | 31,248,787 | 29,880,090 | 95.62 | 33.18 |
| Chromosome 7 | 29,697,621 | 28,248,377 | 95.12 | 32.08 |
| Chromosome 8 | 28,443,022 | 26,895,722 | 94.56 | 32.08 |
| Chromosome 9 | 23,012,720 | 21,770,033 | 94.6 | 34.67 |
| Chromosome 10 | 23,207,287 | 22,091,016 | 95.19 | 38.46 |
| Chromosome 11 | 29,021,106 | 26,504,976 | 91.33 | 30.01 |
| Chromosome 12 | 27,531,856 | 25,406,397 | 92.28 | 32.52 |
| Total/Average | 373,245,519 | 356,446,902 | 95.5 | 33.46 |
| ^a^Length of Nipponbare refenrence genome (Genbank accession - PRJDB1747). | | |  |  |
| ^b^Length of consensus sequence. | |  |  |  |
| ^c^Coverage with locations containing at least one read. | | |  |  |
| ^d^Sequencing depth of reads. | |  |  |  |

| Table S2e: Coverage of the reads from *japonica* 14 to nuclear Nipponbare reference genome | | | |  |
| --- | --- | --- | --- | --- |
| Chromosome | Chromosome length (bp) ^a^ | Aligned length (bp) ^b^ | Coverage in breadth (%)^c^ | Depth^d^ |
| Chromosome 1 | 43,270,923 | 42,288,673 | 97.73 | 40.51 |
| Chromosome 2 | 35,937,250 | 35,196,943 | 97.94 | 41.11 |
| Chromosome 3 | 36,413,819 | 35,845,763 | 98.44 | 39.68 |
| Chromosome 4 | 35,502,694 | 33,951,226 | 95.63 | 41.65 |
| Chromosome 5 | 29,958,434 | 29,470,112 | 98.37 | 40.5 |
| Chromosome 6 | 31,248,787 | 30,198,828 | 96.64 | 39.91 |
| Chromosome 7 | 29,697,621 | 28,515,656 | 96.02 | 38.27 |
| Chromosome 8 | 28,443,022 | 27,242,726 | 95.78 | 38.98 |
| Chromosome 9 | 23,012,720 | 22,057,692 | 95.85 | 39.98 |
| Chromosome 10 | 23,207,287 | 22,450,729 | 96.74 | 47.84 |
| Chromosome 11 | 29,021,106 | 26,978,020 | 92.96 | 36.29 |
| Chromosome 12 | 27,531,856 | 25,954,281 | 94.27 | 41.54 |
| Total/Average | 373,245,519 | 360,150,649 | 96.49 | 40.41 |
| ^a^Length of Nipponbare refenrence genome (Genbank accession - PRJDB1747). | | |  |  |
| ^b^Length of consensus sequence. | |  |  |  |
| ^c^Coverage with locations containing at least one read. | |  |  |  |
| ^d^Sequencing depth of reads. | |  |  |  |

| Table S3a. Mapping and coverage of the reads from landraces to mitochondrial Nipponbare reference genome (Genbank accession: BA00029.3) | | | | | |
| --- | --- | --- | --- | --- | --- |
| Lanraces | Total reads | Mapped reads | Percentage of mapped reads | Mean mapping quality | Coverage |
| *indica* 12 | 129,251,696 | 2,118,312 | 1.64% | 26.26 | 94.39% |
| *indica* 13 | 112,867,645 | 2,628,186 | 2.33% | 26.83 | 94.10% |
| *indica* 15 | 151,576,754 | 2,931,913 | 1,93% | 26.42 | 95.63% |
| *japonica* 11 | 133,602,832 | 2,045,302 | 1.53% | 26.91 | 99.91% |
| *japonica* 14 | 161,141,384 | 3,328,525 | 2.07% | 26.81 | 99.93% |

| Table S3b: Mapping and coverage of the reads from landraces to chloroplast Nipponbare reference genome (Genbank accession: NC_001320.1) | | | | | |
| --- | --- | --- | --- | --- | --- |
| Lines | Total reads | Mapped reads | Percentage of mapped reads | Mean mapping quality | Coverage |
| *indica* 12 | 129,251,696 | 4,713,284 | 3.65% | 38.66 | 100% |
| *indica* 13 | 112,867,645 | 4,260,434 | 3.77% | 39.62 | 100% |
| *indica* 15 | 151,576,754 | 5,103,889 | 3.37% | 38.58 | 100% |
| *japonica* 11 | 133,602,832 | 4,975,528 | 3.72% | 39.38 | 100% |
| *japonica* 14 | 161,141,384 | 8,073,088 | 5.01% | 39.29 | 100% |

| Table S4a: The number of common reads mapped to both chromosome and organelle of the reference genome in *indica* 12 | | | | |
| --- | --- | --- | --- | --- |
| Chromosome | Mitochondria | Coverage in breadth | Chloroplast | Coverage in breadth |
| Chromosome 1 | 227,490 | 0.19% | 488,345 | 0.22% |
| Chromosome 2 | 73,220 | 0.06% | 360,390 | 0.21% |
| Chromosome 3 | 44,493 | 0.09% | 128,826 | 0.12% |
| Chromosome 4 | 204,491 | 0.16% | 1,014,418 | 0.43% |
| Chromosome 5 | 33,003 | 0.05% | 154,329 | 0.15% |
| Chromosome 6 | 55,853 | 0.14% | 239,920 | 0.16% |
| Chromosome 7 | 17,558 | 0.03% | 152,844 | 0.11% |
| Chromosome 8 | 37,731 | 0.05% | 343,474 | 0.20% |
| Chromosome 9 | 136,807 | 0.21% | 73,153 | 0.16% |
| Chromosome 10 | 161,099 | 0.19% | 1,300,226 | 0.78% |
| Chromosome 11 | 5,128 | 0.04% | 9,221 | 0.05% |
| Chromosome 12 | 660,889 | 1% | 379,264 | 0.31% |
| Total/Average | 1,657,762 | 0.17% | 4,644,410 | 0.23% |

| Table S4b: The number of common reads mapped to both chromosome and organelle of the reference genome in *indica* 13 | | | | |
| --- | --- | --- | --- | --- |
| Chromosome | Mitochondria | Coverage in breadth | Chloroplast | Coverage in breadth |
| Chromosome 1 | 282,386 | 0.19% | 449,514 | 0.23% |
| Chromosome 2 | 72,683 | 0.06% | 339,119 | 0.21% |
| Chromosome 3 | 55,556 | 0.10% | 110,052 | 0.13% |
| Chromosome 4 | 226,622 | 0.17% | 917,755 | 0.44% |
| Chromosome 5 | 32,271 | 0.06% | 129,983 | 0.16% |
| Chromosome 6 | 64,928 | 0.15% | 220,752 | 0.17% |
| Chromosome 7 | 17,508 | 0.04% | 138,564 | 0.12% |
| Chromosome 8 | 37,539 | 0.06% | 310,697 | 0.21% |
| Chromosome 9 | 181,973 | 0.22% | 65,055 | 0.12% |
| Chromosome 10 | 162,276 | 0.20% | 1,174,585 | 0.79% |
| Chromosome 11 | 7,365 | 0.04% | 8,684 | 0.05% |
| Chromosome 12 | 899,088 | 0.98% | 354,850 | 0.31% |
| Total/Average | 2,040,195 | 0.18% | 4,219,610 | 0.24% |

| Table S4c: The number of common reads mapped to both chromosome and organelle of the reference genome in *indica* 15 | | | | |
| --- | --- | --- | --- | --- |
| Chromosome | Mitochondria | Coverage in breadth | Chloroplast | Coverage in breadth |
| Chromosome 1 | 307,780 | 0.19% | 526,537 | 0.23% |
| Chromosome 2 | 83,636 | 0.07% | 392,191 | 0.21% |
| Chromosome 3 | 58,707 | 0.10% | 138,391 | 0.12% |
| Chromosome 4 | 252,267 | 0.16% | 1,089,067 | 0.43% |
| Chromosome 5 | 37,314 | 0.05% | 161,180 | 0.15% |
| Chromosome 6 | 70,861 | 0.15% | 264,385 | 0.16% |
| Chromosome 7 | 20,675 | 0.03% | 165,438 | 0.12% |
| Chromosome 8 | 44,172 | 0.05% | 373,032 | 0.21% |
| Chromosome 9 | 194,467 | 0.22% | 80,781 | 0.12% |
| Chromosome 10 | 181,837 | 0.20% | 1,401,223 | 0.78% |
| Chromosome 11 | 7,588 | 0.04% | 9,797 | 0.04% |
| Chromosome 12 | 975,767 | 1.02% | 421,290 | 0.31% |
| Total/Average | 2,235,071 | 0.18% | 5,023,312 | 0.23% |

| Table S4d: The number of common reads mapped to both chromosome and organelle of the reference genome in *japonica* 11 | | | | |
| --- | --- | --- | --- | --- |
| Chromosome | Mitochondria | Coverage in breadth | Chloroplast | Coverage in breadth |
| Chromosome 1 | 210,134 | 0.18% | 519,509 | 0.22% |
| Chromosome 2 | 77,469 | 0.06% | 406,647 | 0.21% |
| Chromosome 3 | 43,645 | 0.09% | 142,517 | 0.11% |
| Chromosome 4 | 191,033 | 0.16% | 1,065,574 | 0.43% |
| Chromosome 5 | 33,547 | 0.05% | 172,005 | 0.14% |
| Chromosome 6 | 59,595 | 0.14% | 251,247 | 0.15% |
| Chromosome 7 | 18,782 | 0.03% | 168,634 | 0.11% |
| Chromosome 8 | 40,587 | 0.05% | 352,918 | 0.20% |
| Chromosome 9 | 124,089 | 0.21% | 77,456 | 0.11% |
| Chromosome 10 | 166,093 | 0.19% | 1,370,980 | 0.78% |
| Chromosome 11 | 5,035 | 0.04% | 9,347 | 0.05% |
| Chromosome 12 | 632,614 | 1.06% | 378,536 | 0.31% |
| Total/Average | 1,602,623 | 0.18% | 4,915,370 | 0.23% |

| Table S4e. The number of common reads mapped to both chromosome and organelle of the reference genome in *japonica* 14 | | | | |
| --- | --- | --- | --- | --- |
| Chromosome | Mitochondria | Coverage in breadth | Chloroplast | Coverage in breadth |
| Chromosome 1 | 340,834 | 0.18% | 838,312 | 0.26% |
| Chromosome 2 | 125,054 | 0.06% | 659,561 | 0.24% |
| Chromosome 3 | 68,948 | 0.09% | 228,888 | 0.15% |
| Chromosome 4 | 307,405 | 0.16% | 1,713,560 | 0.46% |
| Chromosome 5 | 53,656 | 0.05% | 274,980 | 0.17% |
| Chromosome 6 | 91,915 | 0.14% | 413,009 | 0.18% |
| Chromosome 7 | 30,680 | 0.03% | 273,088 | 0.14% |
| Chromosome 8 | 66,205 | 0.05% | 573,412 | 0.24% |
| Chromosome 9 | 218,666 | 0.21% | 128,960 | 0.15% |
| Chromosome 10 | 269,280 | 0.19% | 2,217,099 | 0.81% |
| Chromosome 11 | 8,033 | 0.03% | 14,670 | 0.09% |
| Chromosome 12 | 1,000,447 | 1.03% | 630,458 | 0.36% |
| Total/Average | 2,581,123 | 0.18% | 7,965,997 | 0.26% |

| Table S5a. Polymorphisms of *indica* 12 genome compared to Nipponbare reference | | | | | | | | | | | | | | | | |
| --- | --- | --- | --- | --- | --- | --- | --- | --- | --- | --- | --- | --- | --- | --- | --- | --- |
| Chromosome | | #SNPs | #SNPs/ 100kb | | #InDels | | #InDels/100kb | | #Insertions | | #Insertions/100kb | | #Deletions | | #Deletions/100kb | |
| Chromosome 1 | | 233,111 | 538.7 | | 36,013 | | 83.23 | | 17,262 | | 39.89 | | 18,751 | | 43.33 | |
| Chromosome 2 | | 204,691 | 569.6 | | 30,041 | | 83.59 | | 14,330 | | 39.88 | | 15,711 | | 43.72 | |
| Chromosome 3 | | 200,651 | 551 | | 18,734 | | 51.45 | | 8,996 | | 24.7 | | 9,738 | | 26.74 | |
| Chromosome 4 | | 149,505 | 421.1 | | 29,794 | | 83.92 | | 14,240 | | 40.11 | | 15,554 | | 43.81 | |
| Chromosome 5 | | 147,436 | 492.1 | | 19,777 | | 66.01 | | 9,403 | | 31.39 | | 10,374 | | 34.63 | |
| Chromosome 6 | | 177,397 | 567.7 | | 20,865 | | 66.77 | | 9,905 | | 31.7 | | 10,960 | | 35.07 | |
| Chromosome 7 | | 156,409 | 526.7 | | 23,934 | | 80.59 | | 11,380 | | 38.32 | | 12,554 | | 42.27 | |
| Chromosome 8 | | 151,679 | 533.3 | | 21,028 | | 73.93 | | 9,969 | | 35.05 | | 11,059 | | 38.88 | |
| Chromosome 9 | | 133,830 | 581.5 | | 20,632 | | 89.65 | | 9,970 | | 43.32 | | 10,662 | | 46.33 | |
| Chromosome 10 | | 142,862 | 615.6 | | 18,277 | | 78.76 | | 8,822 | | 38.01 | | 9,455 | | 40.74 | |
| Chromosome 11 | | 167,507 | 577.2 | | 21,073 | | 72.61 | | 10,199 | | 35.14 | | 10,874 | | 37.47 | |
| Chromosome 12 | | 142,749 | 518.5 | | 18,343 | | 66.62 | | 8,761 | | 31.82 | | 9,582 | | 34.8 | |
| Total/Average | | 2,007,827 | 537.9 | | 278,511 | | 74.62 | | 133,237 | | 35.7 | | 145,274 | | 38.92 | |
| Table S5b: Polymorphisms in *indica* 13 genome compared to Nipponbare reference | | | | | | | | | | | | | | | |  |
| Chromosome | #SNPs | | | #SNPs/ 100kb | #InDels | #InDels/100kb | | #Insertions | | #Insertions/100kb | | #Deletions | | #Deletions/100kb | |  |
| Chromosome 1 | 224,298 | | | 518.4 | 34,391 | 79.48 | | 16,566 | | 38.28 | | 17,825 | | 41.19 | |  |
| Chromosome 2 | 189,551 | | | 527.4 | 28,057 | 78.07 | | 13,441 | | 37.4 | | 14,616 | | 40.67 | |  |
| Chromosome 3 | 187,382 | | | 514.6 | 28,010 | 76.92 | | 13,495 | | 37.06 | | 14,515 | | 39.86 | |  |
| Chromosome 4 | 149,779 | | | 421.9 | 20,250 | 57.04 | | 9,518 | | 26.81 | | 10,732 | | 30.23 | |  |
| Chromosome 5 | 142,383 | | | 475.3 | 20,465 | 68.31 | | 9,861 | | 32.92 | | 10,604 | | 35.4 | |  |
| Chromosome 6 | 159,251 | | | 509.6 | 21,601 | 69.13 | | 10,304 | | 32.97 | | 11,297 | | 36.15 | |  |
| Chromosome 7 | 166,327 | | | 560.1 | 22,802 | 76.78 | | 10,845 | | 36.52 | | 11,957 | | 40.26 | |  |
| Chromosome 8 | 142,148 | | | 499.8 | 19,595 | 68.89 | | 9,558 | | 33.6 | | 10,037 | | 35.29 | |  |
| Chromosome 9 | 119,769 | | | 520.4 | 16,377 | 71.16 | | 7,923 | | 34.43 | | 8,454 | | 36.74 | |  |
| Chromosome 10 | 136,518 | | | 588.3 | 17,831 | 76.83 | | 8,450 | | 36.41 | | 9,381 | | 40.42 | |  |
| Chromosome 11 | 161,660 | | | 557 | 20,944 | 72.17 | | 10,168 | | 35.04 | | 10,776 | | 37.13 | |  |
| Chromosome 12 | 135,086 | | | 490.7 | 18,077 | 65.66 | | 8,803 | | 31.97 | | 9,274 | | 33.68 | |  |
| Total/Average | 1,914,152 | | | 512.8 | 268,400 | 71.91 | | 128,932 | | 34.54 | | 139,468 | | 37.37 | |  |

| Table S5c: Polymorphisms in *indica* 15 genome compared to Nipponbare reference | | | | | | | | | | | | | | | | |  |
| --- | --- | --- | --- | --- | --- | --- | --- | --- | --- | --- | --- | --- | --- | --- | --- | --- | --- |
| Chromosome | | #SNPs | | #SNPs/ 100kb | | #InDels | | #InDels/100kb | | #Insertions | | #Insertions/100kb | | #Deletions | | #Deletions/100kb |  |
| Chromosome 1 | | 263,191 | | 608.2 | | 39,896 | | 92.2 | | 19,079 | | 44.09 | | 20,817 | | 48.11 |  |
| Chromosome 2 | | 221,344 | | 615.9 | | 32,123 | | 89.39 | | 15,309 | | 42.6 | | 16,814 | | 46.79 |  |
| Chromosome 3 | | 219,847 | | 603.7 | | 32,136 | | 88.25 | | 15,299 | | 42.01 | | 16,837 | | 46.24 |  |
| Chromosome 4 | | 181,547 | | 511.4 | | 23,641 | | 66.59 | | 11,161 | | 31.44 | | 12,480 | | 35.15 |  |
| Chromosome 5 | | 164,420 | | 548.8 | | 21,965 | | 73.32 | | 10,534 | | 35.16 | | 11,431 | | 38.16 |  |
| Chromosome 6 | | 183,357 | | 586.8 | | 24,111 | | 77.16 | | 11,471 | | 36.71 | | 12,640 | | 40.45 |  |
| Chromosome 7 | | 180,581 | | 608.1 | | 23,957 | | 80.67 | | 11,324 | | 38.13 | | 12,633 | | 42.54 |  |
| Chromosome 8 | | 171,050 | | 601.4 | | 21,968 | | 77.24 | | 10,457 | | 36.76 | | 11,511 | | 40.47 |  |
| Chromosome 9 | | 142,183 | | 617.8 | | 18,946 | | 82.33 | | 9,050 | | 39.33 | | 9,896 | | 43 |  |
| Chromosome 10 | | 155,806 | | 671.4 | | 19,576 | | 84.35 | | 9,266 | | 39.93 | | 10,310 | | 44.43 |  |
| Chromosome 11 | | 192,245 | | 662.4 | | 23,848 | | 82.17 | | 11,471 | | 39.53 | | 12,377 | | 42.65 |  |
| Chromosome 12 | | 165,847 | | 602.4 | | 20,872 | | 75.81 | | 10,008 | | 36.35 | | 10,864 | | 39.46 |  |
| Total/Average | | 2,241,418 | | 600.5 | | 303,039 | | 81.19 | | 144,429 | | 38.7 | | 158,610 | | 42.49 |  |
| Table S5d: Polymorphisms in *japonica* 11 genome compared to Nipponbare reference | | | | | | | | | | | | | | | | | |
| Chromosome | #SNPs | | #SNPs/ 100kb | | #InDels | | #InDels/100kb | | #Insertions | | #Insertions/100kb | | #Deletions | | #Deletions/100kb | | |
| Chromosome 1 | 76,970 | | 177.9 | | 13,444 | | 31.07 | | 6,565 | | 15.17 | | 6,879 | | 15.9 | | |
| Chromosome 2 | 43,433 | | 120.9 | | 7,676 | | 21.36 | | 3,608 | | 10.04 | | 4,068 | | 11.32 | | |
| Chromosome 3 | 51,767 | | 142.2 | | 8,204 | | 22.53 | | 3,903 | | 10.72 | | 4,301 | | 11.81 | | |
| Chromosome 4 | 69,800 | | 196.6 | | 10,664 | | 30.04 | | 5,084 | | 14.32 | | 5,580 | | 15.72 | | |
| Chromosome 5 | 44,144 | | 147.4 | | 6,733 | | 22.47 | | 3,327 | | 11.11 | | 3,406 | | 11.37 | | |
| Chromosome 6 | 54,649 | | 174.9 | | 8,306 | | 26.58 | | 4,003 | | 12.81 | | 4,303 | | 13.77 | | |
| Chromosome 7 | 54,984 | | 185.1 | | 8,348 | | 28.11 | | 4,047 | | 13.63 | | 4,301 | | 14.48 | | |
| Chromosome 8 | 82,605 | | 290.4 | | 10,527 | | 37.01 | | 5,121 | | 18 | | 5,406 | | 19.01 | | |
| Chromosome 9 | 55,548 | | 241.4 | | 7,148 | | 31.06 | | 3,413 | | 14.83 | | 3,735 | | 16.23 | | |
| Chromosome 10 | 55,407 | | 238.7 | | 7,245 | | 31.22 | | 3,374 | | 14.54 | | 3,871 | | 16.68 | | |
| Chromosome 11 | 70,847 | | 244.1 | | 9,878 | | 34.04 | | 4,881 | | 16.82 | | 4,997 | | 17.22 | | |
| Chromosome 12 | 79,500 | | 288.8 | | 10,015 | | 36.38 | | 4,704 | | 17.09 | | 5,311 | | 19.29 | | |
| Total/Average | 739,654 | | 198.2 | | 108,188 | | 28.99 | | 52,030 | | 13.94 | | 56,158 | | 15.05 | | |

| Table S5e: Polymorphisms in *japonica* 14 genome compared to Nipponbare reference | | | | | | | | |
| --- | --- | --- | --- | --- | --- | --- | --- | --- |
| Chromosome | #SNPs | #SNPs/ 100kb | #InDels | #InDels/100kb | #Insertions | #Insertions/100kb | #Deletions | #Deletions/100kb |
| Chromosome 1 | 70,581 | 163.1 | 11,871 | 27.43 | 5,786 | 13.37 | 6,085 | 14.06 |
| Chromosome 2 | 51,315 | 142.8 | 8,514 | 23.69 | 3,980 | 11.07 | 4,534 | 12.62 |
| Chromosome 3 | 34,881 | 95.8 | 6,324 | 17.37 | 3,045 | 8.362 | 3,279 | 9 |
| Chromosome 4 | 75,940 | 213.9 | 10,667 | 30.05 | 5,102 | 14.37 | 5,565 | 15.67 |
| Chromosome 5 | 37,355 | 124.7 | 5,553 | 18.54 | 2,682 | 8.952 | 2,871 | 9.58 |
| Chromosome 6 | 51,802 | 165.8 | 7,802 | 24.97 | 3,725 | 11.92 | 4,077 | 13.05 |
| Chromosome 7 | 45,843 | 154.4 | 6,936 | 23.36 | 3,398 | 11.44 | 3,538 | 11.91 |
| Chromosome 8 | 77,480 | 272.4 | 9,701 | 34.11 | 4,687 | 16.48 | 5,014 | 17.63 |
| Chromosome 9 | 57,573 | 250.2 | 7,044 | 30.61 | 3,379 | 14.68 | 3,665 | 15.93 |
| Chromosome 10 | 71,795 | 309.4 | 6,455 | 27.81 | 3,019 | 13.01 | 3,436 | 14.81 |
| Chromosome 11 | 63,720 | 219.6 | 8,855 | 30.51 | 4,331 | 14.92 | 4,524 | 15.59 |
| Chromosome 12 | 52,630 | 191.2 | 7,061 | 25.65 | 3,358 | 12.2 | 3,703 | 13.45 |
| Total/Average | 690,915 | 185.1 | 96,783 | 25.93 | 46,492 | 12.46 | 50,291 | 13.47 |

Table S6: List of gene containing high nsSNPs shared in the five Vietnamese landraces

LOC_Os07g09210.1 retrotransposon protein, putative, Ty3-gypsy subclass, expressed

LOC_Os07g30340.1 retrotransposon protein, putative, unclassified

LOC_Os02g12910.1 receptor-like protein kinase 5 precursor, putative, expressed

LOC_Os04g01124.1 retrotransposon protein, putative, unclassified

LOC_Os11g45090.1 NB-ARC domain containing protein, expressed

LOC_Os10g22910.1 expressed protein

LOC_Os11g40840.1 receptor-like protein kinase 2 precursor, putative, expressed

LOC_Os10g03230.1 expressed protein

LOC_Os01g11640.1 expressed protein

LOC_Os10g30820.1 expressed protein

LOC_Os11g38780.1 EF hand family protein, putative

LOC_Os06g38270.1 retrotransposon protein, putative, Ty3-gypsy subclass, expressed

LOC_Os12g10110.1 retrotransposon protein, putative, unclassified, expressed

LOC_Os06g37520.1 zinc knuckle domain containing protein, expressed

LOC_Os08g07710.1 hypothetical protein

LOC_Os10g07624.1 retrotransposon protein, putative, unclassified, expressed

LOC_Os12g30140.1 retrotransposon protein, putative, unclassified

LOC_Os08g41660.1 expressed protein

LOC_Os09g08490.1 seven-transmembrane-domain protein 1, putative, expressed

LOC_Os04g07900.1 expressed protein

LOC_Os01g59400.1 expressed protein

LOC_Os03g05950.1 retrotransposon protein, putative, unclassified, expressed

LOC_Os03g23770.1 retrotransposon protein, putative, unclassified, expressed

LOC_Os08g14510.1 retrotransposon, putative, centromere-specific

LOC_Os07g02570.1 NB-ARC domain containing protein, expressed

LOC_Os10g08210.1 transposon protein, putative, CACTA, En/Spm sub-class, expressed

LOC_Os10g07622.1 retrotransposon protein, putative, unclassified, expressed

LOC_Os11g35000.1 expressed protein

LOC_Os09g04760.1 hypothetical protein

LOC_Os11g38080.1 retrotransposon protein, putative, unclassified

LOC_Os08g17480.1 retrotransposon protein, putative, Ty3-gypsy subclass, expressed

LOC_Os12g16740.1 retrotransposon protein, putative, Ty1-copia subclass, expressed

LOC_Os12g08860.1 expressed protein

LOC_Os11g30880.1 retrotransposon protein, putative, unclassified, expressed

LOC_Os09g03520.1 retrotransposon, putative, centromere-specific

LOC_Os08g17460.1 retrotransposon protein, putative, unclassified

LOC_Os07g16380.1 expressed protein

LOC_Os04g01190.1 retrotransposon protein, putative, unclassified, expressed

LOC_Os07g13230.1 eukaryotic initiation factor 5A hypusine, DNA-binding OB fold family protein, expressed

LOC_Os02g34030.1 retrotransposon protein, putative, unclassified, expressed

LOC_Os12g13680.1 hypothetical protein

LOC_Os04g53160.1 NBS-LRR disease resistance protein, putative, expressed

LOC_Os12g14910.1 hypothetical protein

LOC_Os07g44220.1 expressed protein

LOC_Os06g08700.1 retrotransposon protein, putative, unclassified

LOC_Os11g41330.1 expressed protein

LOC_Os07g45120.1 expressed protein

LOC_Os09g10560.1 expressed protein

LOC_Os09g02580.1 hypothetical protein

LOC_Os03g36200.1 retrotransposon protein, putative, unclassified

LOC_Os08g19710.1 retrotransposon protein, putative, Ty3-gypsy subclass, expressed

LOC_Os09g03670.1 retrotransposon protein, putative, unclassified, expressed

LOC_Os04g03210.1 receptor kinase, putative, expressed

LOC_Os04g01200.1 retrotransposon protein, putative, Ty3-gypsy subclass, expressed

LOC_Os01g41770.1 leucine rich repeat protein, putative, expressed

LOC_Os09g10310.1 retrotransposon protein, putative, Ty3-gypsy subclass

LOC_Os12g17710.1 expressed protein

LOC_Os10g08190.1 transposon protein, putative, CACTA, En/Spm sub-class, expressed

LOC_Os07g16640.1 retrotransposon protein, putative, unclassified

LOC_Os10g08430.1 hypothetical protein

LOC_Os09g01110.1 retrotransposon protein, putative, Ty3-gypsy subclass, expressed

LOC_Os01g26300.1 OsWAK9 - OsWAK receptor-like cytoplasmic kinase OsWAK-RLCK, expressed

LOC_Os09g26720.1 retrotransposon protein, putative, unclassified

LOC_Os09g12580.1 expressed protein

LOC_Os10g22020.1 RGH2B, putative, expressed

LOC_Os09g08270.1 nodulin MtN3 family protein, putative, expressed

LOC_Os01g37380.1 transposon protein, putative, unclassified, expressed

LOC_Os06g42870.1 transposon protein, putative, Pong sub-class, expressed

LOC_Os12g13700.1 hypothetical protein

LOC_Os06g35220.1 retrotransposon protein, putative, Ty3-gypsy subclass, expressed

LOC_Os09g14420.1 expressed protein

LOC_Os12g07410.1 retrotransposon protein, putative, unclassified, expressed

LOC_Os03g15130.1 expressed protein

LOC_Os06g35250.1 retrotransposon protein, putative, Ty3-gypsy subclass, expressed

LOC_Os01g11140.1 retrotransposon protein, putative, unclassified, expressed

LOC_Os04g05290.1 retrotransposon protein, putative, Ty3-gypsy subclass, expressed

LOC_Os04g21070.1 retrotransposon protein, putative, unclassified

LOC_Os12g27700.1 retrotransposon protein, putative, Ty3-gypsy subclass, expressed

LOC_Os07g24080.1 retrotransposon, putative, centromere-specific, expressed

LOC_Os02g30030.1 retrotransposon protein, putative, Ty3-gypsy subclass, expressed

LOC_Os11g36860.1 retrotransposon protein, putative, unclassified, expressed

LOC_Os10g33080.1 leucine-rich repeat receptor protein kinase EXS precursor, putative, expressed

LOC_Os08g17340.1 hypothetical protein

LOC_Os03g62388.1 expressed protein

LOC_Os01g06160.1 retrotransposon protein, putative, Ty3-gypsy subclass, expressed

LOC_Os04g07600.1 AGAP002737-PA, putative, expressed

LOC_Os05g13460.1 retrotransposon protein, putative, unclassified, expressed

LOC_Os10g33000.1 retrotransposon protein, putative, unclassified, expressed

LOC_Os04g53120.1 NB-ARC domain containing protein, expressed

LOC_Os04g14160.1 retrotransposon protein, putative, unclassified

LOC_Os11g21900.1 retrotransposon protein, putative, unclassified, expressed

LOC_Os11g31320.1 retrotransposon protein, putative, unclassified

LOC_Os08g17740.1 transposon protein, putative, CACTA, En/Spm sub-class, expressed

LOC_Os07g12820.1 B3 DNA binding domain containing protein

LOC_Os10g07986.1 expressed protein

LOC_Os03g36220.1 retrotransposon protein, putative, unclassified, expressed

LOC_Os12g19490.1 retrotransposon protein, putative, unclassified, expressed

LOC_Os01g06570.1 expressed protein

LOC_Os04g01100.1 transposon protein, putative, unclassified

LOC_Os08g20740.1 expressed protein

LOC_Os11g28790.1 retrotransposon protein, putative, unclassified, expressed

LOC_Os01g06200.1 retrotransposon protein, putative, unclassified, expressed

LOC_Os05g12720.1 conserved hypothetical protein

LOC_Os09g25280.1 retrotransposon protein, putative, LINE subclass, expressed

LOC_Os08g19740.1 retrotransposon protein, putative, Ty1-copia subclass

LOC_Os11g37010.1 expressed protein

LOC_Os08g14290.1 retrotransposon protein, putative, Ty3-gypsy subclass, expressed

LOC_Os08g17240.1 retrotransposon, putative, centromere-specific

LOC_Os10g08760.1 hypothetical protein

LOC_Os02g24090.1 retrotransposon protein, putative, unclassified, expressed

LOC_Os01g61140.1 retrotransposon protein, putative, unclassified, expressed

LOC_Os12g34500.1 expressed protein

LOC_Os04g25810.1 retrotransposon protein, putative, unclassified

LOC_Os11g43500.1 NBS-LRR type disease resistance protein, putative, expressed

LOC_Os04g01380.1 retrotransposon protein, putative, Ty3-gypsy subclass, expressed

LOC_Os11g28570.1 retrotransposon protein, putative, Ty3-gypsy subclass, expressed

LOC_Os09g24190.1 expressed protein

LOC_Os05g30200.1 expressed protein

LOC_Os06g07260.1 OsWAK62 - OsWAK short gene, expressed

LOC_Os10g13390.1 retrotransposon protein, putative, Ty3-gypsy subclass, expressed

LOC_Os09g09580.1 retrotransposon protein, putative, Ty3-gypsy subclass, expressed

LOC_Os10g32050.1 ankyrin repeat domain containing protein, expressed

LOC_Os08g17490.1 retrotransposon protein, putative, Ty3-gypsy subclass, expressed

LOC_Os09g16750.1 retrotransposon protein, putative, unclassified, expressed

LOC_Os06g35400.1 hypothetical protein

LOC_Os07g40210.1 retrotransposon protein, putative, LINE subclass, expressed

LOC_Os06g15750.1 NB-ARC domain containing protein, expressed

LOC_Os11g27100.1 retrotransposon protein, putative, unclassified, expressed

LOC_Os08g20110.1 retrotransposon protein, putative, Ty3-gypsy subclass, expressed

LOC_Os09g04970.1 retrotransposon protein, putative, Ty3-gypsy subclass, expressed

LOC_Os07g16830.1 retrotransposon protein, putative, Ty3-gypsy subclass, expressed

LOC_Os10g24290.1 retrotransposon protein, putative, unclassified, expressed

LOC_Os06g50200.1 retrotransposon protein, putative, unclassified, expressed

LOC_Os03g36239.1 retrotransposon protein, putative, Ty3-gypsy subclass, expressed

LOC_Os02g30880.1 retrotransposon protein, putative, unclassified, expressed

LOC_Os10g09640.1 retrotransposon, putative, centromere-specific

LOC_Os09g27300.1 retrotransposon protein, putative, unclassified, expressed

LOC_Os08g21390.1 retrotransposon, putative, centromere-specific

LOC_Os10g09970.1 retrotransposon protein, putative, Ty3-gypsy subclass, expressed

LOC_Os08g20160.1 SWIM zinc finger family protein, putative, expressed

LOC_Os09g06710.1 transposon protein, putative, CACTA, En/Spm sub-class, expressed

LOC_Os03g29520.1 retrotransposon protein, putative, unclassified, expressed

LOC_Os11g30300.1 expressed protein

LOC_Os04g04890.1 retrotransposon protein, putative, Ty3-gypsy subclass, expressed

LOC_Os09g11220.1 retrotransposon protein, putative, unclassified, expressed

LOC_Os11g07630.1 hypothetical protein

LOC_Os08g28720.1 retrotransposon protein, putative, unclassified

LOC_Os04g08784.1 retrotransposon protein, putative, unclassified, expressed

LOC_Os03g26880.1 retrotransposon protein, putative, Ty3-gypsy subclass, expressed
